# An Applied Statistics dataset for human vs AI-generated answer classification

**DOI:** 10.1016/j.dib.2024.110240

**Published:** 2024-03-02

**Authors:** Md. Shahidul Salim, Sk Imran Hossain

**Affiliations:** Khulna University of Engineering & Technology, Khulna 9203, Bangladesh

**Keywords:** LLM, Assignment, Transformer, AI

## Abstract

Due to the increasing popularity of Large Language Models (LLMs) like ChatGPT, students from various fields now commonly rely on AI-powered text generation tools to complete their assignments. This poses a challenge for course instructors who struggle to identify the authenticity of submitted work. Several AI detection tools for differentiating human-generated text from AI-generated text exist for domains like medical and coding, and available generic tools do not perform well on domain-specific tasks. Those AI detection tools depend on LLM, and to train the LLM, an instruction dataset is needed that helps the LLM to learn the differences between patterns of human-generated text and AI-generated text. To help with the creation of a tool for Applied Statistics, we have created a dataset containing 4231 question-and-answer combinations. To create the dataset, first, we collected 116 questions covering a wide range of topics from Applied Statistics selected by domain experts. Second, we created a framework to randomly distribute and collect answers to the questions from students. Third, we collected answers to fifty assigned questions from each of the 100 students participating in the work. Fourth, we generated an equal number of AI-generated answers using ChatGPT. The prepared dataset will be useful for creating AI-detector tools for the Applied Statistics domain as well as benchmarking AI-detector tools, and the proposed data preparation framework will be useful for collecting data for other domains.

Specifications Table

**Table:** 

Subject	Artificial Intelligence
Specific subject area	Natural Language Processing, LLM, Transformer
Data format	Raw
Type of data	Text
Data collection	This dataset was obtained from the assignments done by 100 students enrolled in undergraduate level Applied Statistics course. First, 116 questions were selected by a domain expert and randomly distributed to the students. Second, each student responded to fifty questions. Finally, the same number of answers were generated using ChatGPT [1]. A custom-built web platform was used to distribute questions and collect answers from the students.
Data source location	Institution: Khulna University of Engineering & Technology City/Town/Region: Khulna Country: Bangladesh
Data accessibility	Repository name: Mendeley Data Data identification number: 10.17632/mh892rksk2 Direct URL to data: https://data.mendeley.com/datasets/mh892rksk2 Instructions for accessing these data: The dataset is publicly accessible using the provided direct URL.

## 1 Value of the Data

• This dataset contains 4231 question-and-answer combinations for 116 Applied Statistics questions selected by domain experts and the answers were generated by 100 human participants and an AI model.
• This dataset can be used to train LLM for Human vs AI-generated answer classification related to Applied Statistics questions.
• This dataset will be useful for the proper benchmarking of AI-detector tools.
• The proposed data collection framework can be used for creating datasets related to different subject domains.

## 2 Data Description

**Fig. 1:**
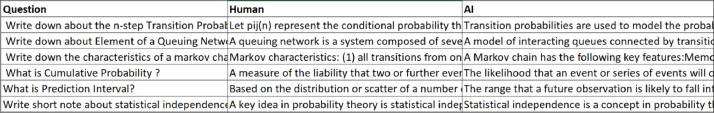
Data sample in Excel file format.

**Fig. 2:**
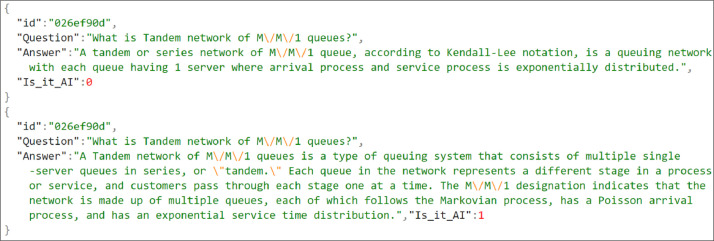
Data sample in JSON list format.

**Fig. 3:**
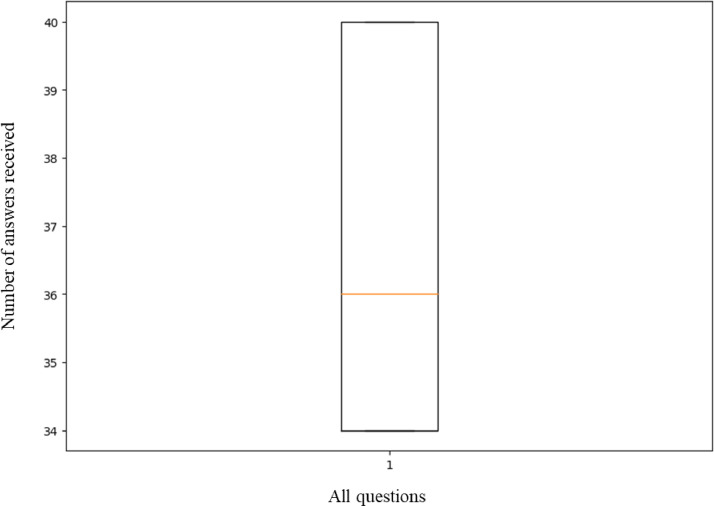
Boxplot showing the number of answers received for all the questions.

The dataset, containing responses to 116 questions from both human and AI sources, is publicly available in a data repository [2]. The dataset is organized into a folder named "AI classifier dataset," which includes 100 Excel files and one JSON list file called *dataset.jsonl*. Each Excel file corresponds to the data collected from one student participating in the study. The file names are randomized to keep the students' identities anonymous. An Excel file consists of three attributes: Question, Human, and AI. Each row of an Excel file represents a question, an answer to that question from a student, and an AI-generated answer collected by the students as shown in Fig. 1. Again, data from 100 Excel files are organized in a single file named *dataset.jsonl* using The JSON file format for ease of use. The *dataset.jsonl* contains four attributes for each entry: an ID, the original question, the answer, and Is_it_AI. A value of 1 for the attribute Is_it_AI represents an AI-generated answer, while 0 represents a human-generated answer. Fig. 2 shows some same entries from the JSON list file. In total, the JSON list file contains 4231 rows of data. In our dataset, number of answers received for each question ranges from 34 to 40. Some variations have occurred because some answers received fewer and duplicate answers from the student. Also, some inappropriate answers were removed. The final summary of the distribution is given in the boxplot shown in Fig. 3. It is a five-number summary of the data: Min: 34.000 Q1: 34.000 Median: 36.000 Q3: 40.000 Max: 40.000. The diversity of the answers is shown in Table 1. For AI answers, the number of unique words is 10,318 and the average length of the answers (in words) is 151.2. For human answers, there are 11,801 unique words, and the average length of the answers (in words) is 77.3. These findings suggest that, on average, AI responses are longer than those from humans. Furthermore, compared to AI responses, human responses show a wider range of unique terms, indicating a possibly higher diversity in vocabulary usage.

**Table 1:** Diversity of the dataset.

Generated answers	Number of unique words	Average length of answers (in words)
AI	10318	151.2
Human	11801	77.3

## 3 Experimental Design, Materials and Methods

**Fig. 4:**
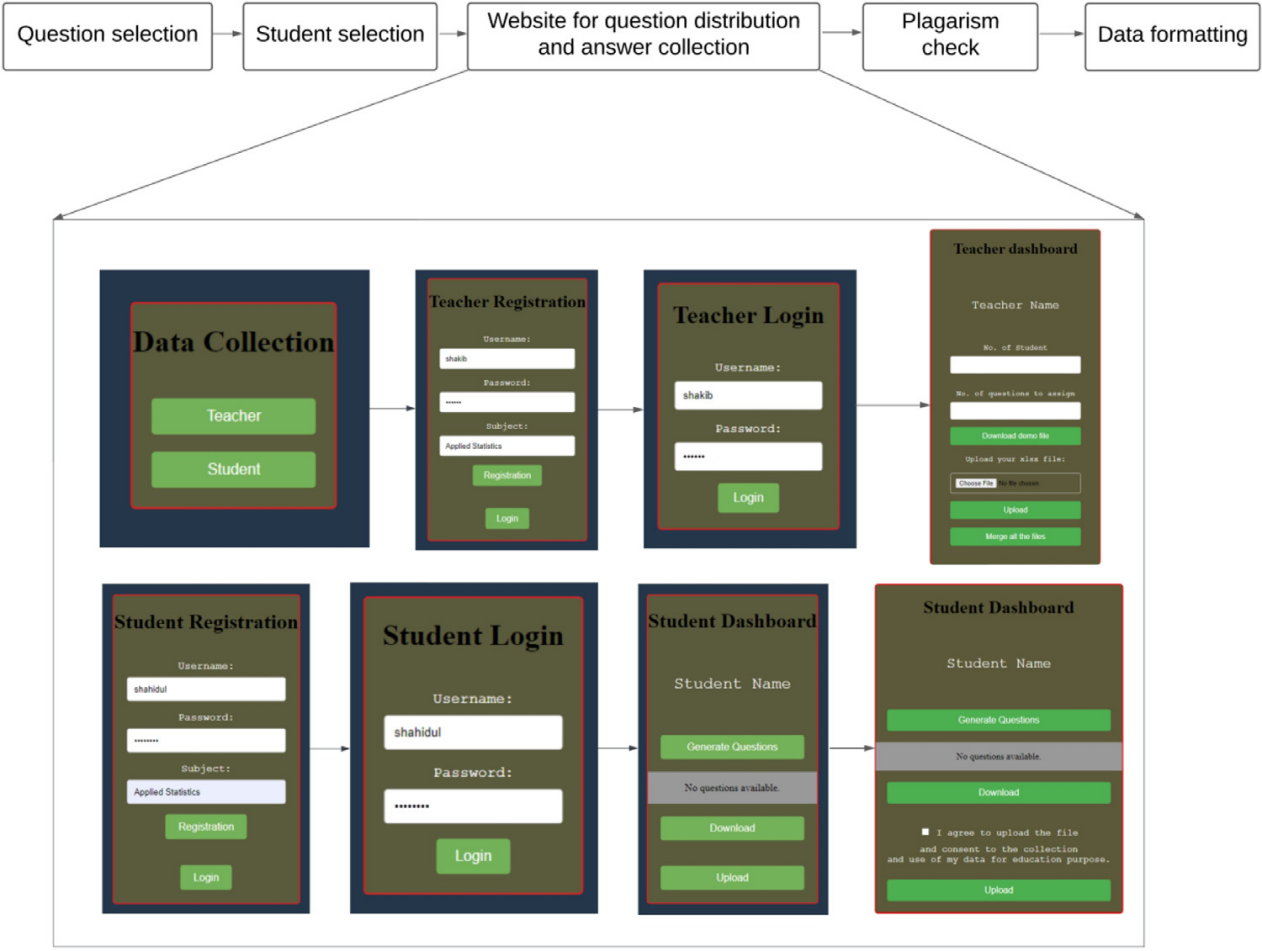
Data collection framework.

Generic AI-detector tools do not perform well on domain-specific tasks [[3], [4], [5]], and preparing a subject-specific human-annotated dataset is tedious. To ease the process of preparing subject-specific datasets, we have created a data collection framework as shown in Fig. 4. The steps involved choosing subjects and questions, selecting students, distributing questions randomly, creating a user interface for answer collection, conducting plagiarism checks, and formatting the gathered data.

First, we need to select the subject and questions for the subject. The subject selected for this work was Applied Statistics mainly because of the available students enrolled in the Applied Statistics course. 116 questions were prepared by two subject experts to ensure that the questions cover a wide range of topics. The experts have Ph.D. degrees and have been teaching this subject at undergraduate and postgraduate levels for more than five years. The teacher selected the questions to cover a wide range of topics from applied statistics.

Second, we need to select students for the task of answering questions manually and also to collect AI-generated answers. We asked a total of 120 students and 100 of them volunteered for the task. Each student volunteered for this work without financial or academic gain. We also trained several classifiers using the 80-20 train-test split of the dataset and achieved good performance from most of them which further indicates that the students performed their tasks properly. Table 2 shows a comparison of the performance of various transformer models for the detection of written assignments by humans and by AI. This testing result indicates that classifiers can classify the data between humans and AI with an acceptable level of accuracy.

**Table 2:** Classification performance of several models tested on the prepared dataset.

Model name	Accuracy(%)	Precision	Recall	f1-score
Bert-base-uncased [6]	89	0.91	0.90	0.88
DistilBERT base uncased finetuned SST-2 [7]	92	0.92	0.90	0.88
Roberta-base [8]	50	0.29	0.50	0.35
Albert-base-v2 [9]	86	0.84	0.83	0.81
Distilbert-base-uncased [7]	88	0.87	0.87	0.85

Third, we created a website for question distribution and answer collection as shown in Fig. 4. The source code for the website is publicly available in the ‘*source code*’ folder of our data repository [2]. The first page has two sections: Teacher and Student. For the teacher section, the teacher needs to complete registration to enter their questions. In the registration part, we need three pieces of information for registration purposes: Username, Password, and Subject. After completing registration user can log in to the teacher dashboard, which has four sections. In the first section, the teacher can specify the number of questions to be answered by each student. In the second section, the teacher can download a demo template file in Excel file format for assigning the questions. After assigning questions, the teacher uploads the file to the dashboard in the third section, which stores all the questions for this subject. In the fourth section, a teacher can merge all the answers collected from the students and convert those data into a JSON list file. Again, the data collection page has another section for the students. Here, students can register like teachers using a username, password, and subject. After completing the registration, students can log in to the dashboard which has three sections: Generate questions, Download, and Upload. After clicking generate questions button, it shows random questions, and students can download those questions as an Excel file. The Excel file has three attributes: Question, Human, and AI. The students can put their own answer for a question in the Human field and also an answer generated by AI tool in the AI field of the Excel file. To make the dataset balanced, we randomly assigned each question to a similar number of students. We used Eq. (1) to determine how many times each question needs to be assigned.

(1) $$Number\;of\;times\;a\;question\;is\;assigned,\;q_{N}=\frac{S_{N}\times P_{N}}{Q_{N}}$$

In Eq. (1) $S_{N}$ is the total number of students, $P_{N}$ is the number of questions to be assigned per student, and $Q_{N}$ is the total number of unique questions. After answering the question, students need to give their consent about using their data for educational purposes and then a student can upload the Excel file to the dashboard, which further the teacher can accumulate and create a JSON list file.

Fourth, after collecting the data, we checked for plagiarism and removed plagiarised answers provided by the students. We have examined plagiarism because we need to ensure that the responses provided by the students differ from each other and also the AI-generated responses are distinct. We used Turnitin software [10] for plagiarism checking. Finally, we formatted the data in JSON file format for ease of use.

## Limitations

All the students participating in the study are from a single university sharing a similar level of schooling. Involving students from multiple universities can increase diversity. Similarly, all the AI-generated answers are only from ChatGPT.

## Ethics Statement

We took consent from the participating students before collecting data from them.

## CRediT authorship contribution statement

**Md. Shahidul Salim:** Conceptualization, Methodology, Software, Data curation, Writing – original draft. **Sk Imran Hossain:** Supervision, Writing – review & editing, Project administration.

## Data Availability

An Applied Statistics dataset for human vs AI-generated answer classification (Original data) (Mendeley Data).
